# Impact of extended critical care outreach service with consultant input at queens hospital

**DOI:** 10.1186/2197-425X-3-S1-A142

**Published:** 2015-10-01

**Authors:** N Muchoki

**Affiliations:** Barking, Havering and Redbridge University Teaching NHS Trust, Critical Care, Romford, United Kingdom

## Introduction

The Critical Care Outreach services are considered to be an essential part of a hospital wide approach in improving the early identification and management of deteriorating patients[1].

Original recommendations made during the modernisation and improvement programme for Critical Care Services within England in 2000 led to different models of the service introduced but they remained mainly nurse led, with mixed approach to service delivery[1].

## Objective

We aimed to compare the difference in patient outcomes on a outreach service with consultant intensivist versus a nurse led outreach service. The CCOT at Queens hospital expanded in September 2013 to include consultant intensivist and the service also had extended the hours of provision from 8 hours /5 days to 7 days/12 hours.

## Methods

Retrospective cohort study of prospective collected data at a teaching university hospital of adult critical care patients admitted from January to June 2012 and January to June 2014.

Outcomes measured were:

- Emergency admissions to critical care. (from ward areas only)
- Critical care readmission:- early < 48hrs; Late >48hrs
- ICU length of stay
- Patients seen by CCOT before admission
- Number of cardiac arrest calls.

## Results

- 15% reduction on number of Cardiac Arrest calls, (excluding A/E, critical care and theatres).
- 5.7% reduction on Length of Stay with a 75% increase in early discharges
- 121% increase in CCOT visits for critical care admissions.
- 10% increase in Emergency admissions

> Critical care admitted sicker patients with higher acute physiology score with 91% reduction on admissions requiring zero organ support

- 0.9%increase in Critical Care readmission (total emergency admissions)

## Conclusions

In our critical care unit (ICU/ HDU) there has been an increase in the number of occupancy over a period of time (Graph 1). The extension of the critical care outreach service to longer hours and to include a consultant has allowed for early discharges leading to a higher turn over in the number of critical care patients. The reduction in cardiac arrest calls could be attributed to an increase in the ´Not for Resuscitation´ decisions initiated by the consultants as well as an increase in service provision. The total cost effectiveness of the service is an area of future research.

**Figure 1:**
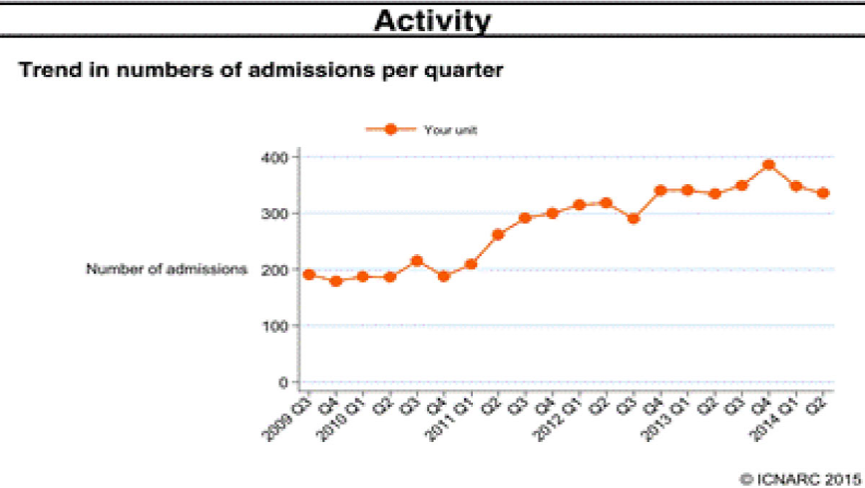
**Activity.**

**Table 1:** Outcomes.

Outcomes	Jan - June 2012	Jan - June 2014
Emergency Admissions	255	281
Critical care re-admissions	7 (<48hrs (early)) 8 (>48hrs (late))	7(<48hrs (early)) 12 (>48hrs (late))
CA calls	116	99
CCOT reviews before admission	87	193
ICU LOS	5.4 days	4.9 days
